# Risk of Potentially Rabid Animal Exposure among Foreign Travelers in Southeast Asia

**DOI:** 10.1371/journal.pntd.0001852

**Published:** 2012-09-27

**Authors:** Watcharapong Piyaphanee, Chatporn Kittitrakul, Saranath Lawpoolsri, Philippe Gautret, Wataru Kashino, Waraluk Tangkanakul, Prangthip Charoenpong, Thitiya Ponam, Suda Sibunruang, Weerapong Phumratanaprapin, Terapong Tantawichien

**Affiliations:** 1 Department of Clinical Tropical Medicine, Faculty of Tropical Medicine, Mahidol University, Bangkok, Thailand; 2 Department of Tropical Hygiene, Faculty of Tropical Medicine, Mahidol University, Bangkok, Thailand; 3 Institut Hospitalo-Universitaire en Maladies Infectieuses et Tropicales, Hôpital Nord, Marseille, France; 4 Suvarnabhumi Port Health Office, Bureau of General Communicable Diseases, Department of Disease Control, Ministry of Public Health, Bang Phli, Samut Prakan, Thailand; 5 Hospital for Tropical Diseases, Faculty of Tropical Medicine, Mahidol University, Bangkok, Thailand; 6 Queen Saovabha Memorial Institute, the Thai Red Cross Society, Bangkok, Thailand; 7 Division of Infectious Diseases, Department of Medicine, Faculty of Medicine, King Chulalongkorn Memorial Hospital, Bangkok, Thailand; Swiss Tropical and Public Health Institute, Switzerland

## Abstract

**Background:**

Each year millions of travelers visit Southeast Asia where rabies is still prevalent. This study aimed to assess the risk of rabies exposure, i.e., by being bitten or licked by an animal, among travelers in Southeast Asia. The secondary objective was to assess their attitudes and practices related to rabies.

**Methodology/Principal Findings:**

Foreign travelers departing to the destination outside Southeast Asia were invited to fill out the study questionnaire in the departure hall of Bangkok International Airport. They were asked about their demographic profile, travel characteristics, pre-travel health preparations, their possible exposure and their practices related to rabies during this trip. From June 2010 to February 2011, 7,681 completed questionnaires were collected. Sixty-two percent of the travelers were male, and the median age was 32 years. 34.0% of the participants were from Western/Central Europe, while 32.1% were from East Asia. Up to 59.3% had sought health information before this trip. Travel clinics were the source of information for 23.6% of travelers. Overall, only 11.6% of the participants had completed their rabies pre-exposure prophylaxis, and 15.3% had received only 1–2 shots, while 73.1% had not been vaccinated at all. In this study, the risk of being bitten was 1.11 per 100 travelers per month and the risk of being licked was 3.12 per 100 travelers per month. Among those who were bitten, only 37.1% went to the hospital to get post exposure treatment. Travelers with East Asian nationalities and longer duration of stay were significantly related to higher risk of animal exposure. Reason for travel was not related to the risk of animal exposure.

**Conclusions:**

Travelers were at risk of being exposed to potentially rabid animals while traveling in Southeast Asia. Many were inadequately informed and unprepared for this life-threatening risk. Rabies prevention advice should be included in every pre-travel visit.

## Author Summary

Rabies is a fatal disease most commonly transmitted through a bite or a lick of a rabid animal on the broken skin. Most deaths from rabies are reported in Asia and Africa where animal rabies is poorly controlled. Not only local people, but travelers in these areas are inevitably at risk also. In this study we surveyed foreign travelers just before they departed Southeast Asia at Bangkok International Airport. We aimed to determine the risk of possible rabies exposure and their attitudes and practices related to rabies. The risk of being bitten among 7,681 participants studied was 1.11 per 100 travelers per month and the risk of being licked was 3.12 per 100 travelers per month. Among those who were bitten, only 37.1% went to the hospital to get rabies post exposure treatment. Travelers with East Asian nationalities and who stay longer were more likely to be exposed to animals. The risk of animal exposure was not related with the reason for travel. These findings confirm that travelers in Southeast Asia were at real risk of possible exposure to rabies. However, most of them were inadequately informed and unprepared for this life-threatening disease. Rabies prevention advice should be given to all travelers in rabies endemic area.

## Introduction

Rabies remains an important neglected disease worldwide. Approximately 50,000–55,000 people die from rabies each year [1]. Although most deaths are reported among local people in high endemic area especially in Asia and Africa [2], travelers in those areas are inevitably at risk if they are bitten by infected animals or if the saliva of an infected animal comes into contact with broken skin or mucosa.

Pre-exposure vaccination is an excellent preventive measure against rabies among travelers. However, it is not routinely recommended to all travelers in endemic areas. Its high price and cost-effectiveness are often debated as discussed in many papers [3]–[6]. Travel medicine practitioners should consider several factors, including the risk of being bitten or licked during trips, rabies endemicity and the availability of medical care at the travel destination and travelers' preferences before recommending a vaccine. Among those factors, the actual risk of animal exposure is thought to be a major one [5], [7], [8].

Southeast Asia is one of the popular tourist destinations for travelers worldwide. Each year, up to 60 million tourists visit Southeast Asia [9], where rabies is still endemic and stray dogs and cats are common. Information regarding the risk of rabies exposure among travelers in Southeast Asia is limited. Therefore, in this study, we aim to determine the incidence and risk factors of possible exposure to rabies, i.e., by being bitten or licked by animals, during their trips in Southeast Asia. The secondary objective was to assess their pre-travel preparation, vaccination rate, knowledge, and practices related to the risk of rabies.

## Methods

This cross-sectional questionnaire based study was conducted in Suvarnabhumi International Airport in Bangkok. Data were collected from adult foreign travelers in the international departure hall. Only travelers who had completed their trip and were departing to the destination outside Southeast Asia were eligible to participate. Travelers of Southeast Asian nationalities or travelers who were just in transit were excluded. The study questionnaire was drafted, tested, and revised before the actual data collection. The final version of the questionnaire comprised of four parts, i.e., general information about the travelers, rabies pre-exposure preparations, knowledge about rabies, and the details of any animal exposure. Animal exposure in this study defined as being bitten or licked by mammals that potentially carry rabies virus. In this study, we considered all licked events were at potential risk of rabies exposure, since most travelers were unsure whether their skin was broken. Apart from English, the questionnaire had been translated into 3 more languages: Chinese, Japanese and Korean.

Data from previous studies showed that approximately 0.69–2.2% of travelers were bitten during their one-month stay in Thailand [10], [11]. Therefore, the sample size was calculated based on the assumed incidence of 1% with confidence interval of 0.75%–1.25%, together with the numbers and nationalities of travelers visiting Thailand in 2008 from Thai Immigration Department. To achieve a 95% confidence level, at least 6,081 travelers were required from all regions.

Since the number of travelers from different continents visiting Thailand were not equally distributed and the majority came from Europe and East Asia. To assure the representativeness of travelers from the different continents, quota sampling was implemented. Therefore, the proportions and numbers of participants required from each continent represented the actual annual travel population to Thailand.

During data collection, the investigator team invited any travelers in the departure hall to participate in the study. Eligible travelers who were willing to participate in the study filled out a questionnaire by themselves. The investigator team was available to help if they needed some assistance or clarification of the questionnaire.

The price per one dose of cell-cultured rabies vaccine in each country was obtained from travel medicine specialists through the EuroTravNet network, from personal communication and from other sources. The mean prices for each country or region was adjusted by using the gross domestic product (GDP) per capita, which were obtained from the World Bank. Then, cost index of rabies vaccine for each country could be calculated (mean price/gross domestic product per capita ×10^4^). In this study, rabies vaccination rate was referred to the percentage of travelers who received any rabies pre-exposure vaccines (3 shots or 1–2 shots) over total number of travelers.

### Statistical analysis

Statistical analysis was conducted using SPSS for Windows, version 10.0.7 (SPSS Inc, Chicago, IL) software.

Continuous data were presented as mean with standard deviation (for normally distributed data), or median with range (for non-normally distributed data). Categorical data were presented as numbers and percentage. The Student t-test was used to compare means of two groups, while the Chi-square test was used for categorical data, as appropriate. Relative risk (RR) and 95% Confidence interval were calculated to determine factors potentially associated with animal exposure and receiving pre-exposure vaccination. Factors with a *p-value* below 0.10 in the univariate models were considered eligible for the multivariate analysis. In this study, a *p-value* of <0.05 was considered as statistically significant.

### Ethics statement

The research protocol as well as the questionnaire was approved by the Ethics Committee of the Faculty of Tropical Medicine, Mahidol University (Approval No. MUTM 2010-015-02). Since this study was a voluntary, anonymous survey among adults and was non-experimental in nature; so the Ethics Committee had waived the written consent and approved to imply that filling the questionnaire represent their consent to participate in this study. All participants were informed of the study's objective and grants verbal consent before filling the questionnaires. No participant-identifiable data was recorded in the questionnaire to maintain confidentiality.

## Results

During the period from June 2010 to February 2011, 7681 questionnaires were collected and analyzed. The sex ratio of males to females of participants was 1.6 and the median age was 32 years. Approximately one third of the participants were from Western/Central Europe and one-third were from East Asia. The main reason for travel was tourism, followed distantly by business and visiting friends and relatives.

Approximately 60% of participants had sought travel health information before the current trip. The most common sources of information were the internet followed by general practitioners, travel clinics, friends and relatives, guidebooks and pharmacists. Only 12% of travelers had completed a course of pre-exposure rabies vaccinations (3 shots) before travel, 15% had received only 1 or 2 shots, while the majority had not been vaccinated for rabies at all. The complete demographic breakdown is shown in Table 1.

**Table 1:** Demographic and travel characteristics (n = 7,681).

	n	%
Sex (n = 7,667)		
Male	4,771	62.2
Female	2,896	37.8
Age (year) [median 32 y; range 17–90 y] (AVR = 35.68 yr)		
17–30	3,529	45.9
31–45	2,422	31.5
46–60	1,307	17.0
≥61	423	5.5
Nationality (n = 7,675)		
Western and Central European	2,612	34.0
East Asian	2,462	32.1
Oceania (Australian, New Zealander)	676	8.8
South Asian	543	7.1
North American	442	5.8
Middle East+Central Asian	330	4.3
Eastern European	256	3.3
Central and South American	180	2.3
African	174	2.3
Reason for travel (n = 7,650)		
Tourism	6,512	85.1
Business	450	5.9
Visiting friends and relatives	420	5.5
Education or research	110	1.4
Other	158	2.1
Had sought any travel health information before leaving (n = 7,628)		
Yes	4,524	59.3
No	3104	40.7
Source of travel heath information*		
Internet	2,417	31.5
General Practitioner	1,892	24.7
Travel Clinic	1,809	23.6
Friends and Relatives	1,249	16.3
Guidebooks/Magazines/News	949	12.4
Pharmacists	875	13.4
Other	87	1.5
Received rabies pre-exposure vaccine		
Complete vaccination (3 shots)	847	11.6
Incomplete vaccination (1–2 shots)	1,121	15.3
No	5,351	73.1

* Travelers could have more than one source of travel health information.

### Travelers' knowledge about rabies and relation to travel clinic visit

Of the 7,681 travelers studied, 1,809 (23.6%) had received pre-travel health advice from a travel clinic; 56% of the travelers in the travel clinic group had received information about rabies, which was significantly higher than travelers who sought pre-travel health advice from other sources (56.0% vs 37.5%, p<0.001). 21% of travelers in the travel clinic group had completed a course of pre-exposure rabies vaccine while only 8% of travelers in non-travel clinic group had completed their prophylaxis (21.4% vs 8.4%, p<0.001).

When the details of traveler knowledge about rabies was analyzed, it was found that most travelers knew that they could get rabies if bitten by an infected animal and that dogs could carry rabies. However, nearly one out of two travelers was not aware that cats could also carry rabies. Moreover, more than one-fourth of travelers thought that the bite of a healthy-looking dog or cat posed no risk of rabies.

Subgroup analysis also revealed that the travelers who had visited a travel clinic possessed some more specific knowledge items than those who did not visit the clinic including that being licked by an animal poses a risk of contracting rabies. The mean knowledge score for those who visited a travel clinic was significantly higher than the score of those who had not received pre-travel health advice from a travel clinic. The details are shown in Table 2.

**Table 2:** Relation of Travel Clinic visit to Travelers' Knowledge about rabies.

	Pre-Travel Preparation	Overall	Visited Travel clinic		*p-value*
			Yes (%)n = 1,809	No (%)n = 5,860	
1	Receive information about rabies	41.90%	56.00%	37.50%	<0.001*
2	Receive complete pre-exposure rabies vaccine (3 doses)	11.6%	21.4%	8.4%	<0.001*

	Knowledge (correct answer)	Overall	Travel Clinic Group (%Know)	Control Group (%Know)	*p-value*
1	You may get rabies if you				
	are bitten by an infected animal (True)	85.30%	85.60%	85.30%	0.769
	are licked by an infected animal on broken skin (True)	40.90%	50.40%	38.00%	<0.001*
	contact with animal's saliva on your normal skin (False)	87.10%	84.50%	87.90%	<0.001*
	eat contaminating food/drinks (False)	90.40%	90.10%	90.50%	0.632
2	Animal that could carries rabies				
	Dog (True)	95.20%	96.70%	94.80%	0.001*
	Cat (True)	53.70%	53.30%	53.90%	0.655
	Snake (False)	96.30%	96.20%	96.40%	0.648
	Chicken (False)	94.30%	94.50%	94.20%	0.533
	Bat (True)	29.50%	41.20%	25.80%	<0.001*
	Monkey (True)	43.30%	55.30%	39.60%	<0.001*
3	If you have already had complete course (3 shots) of rabies vaccine before travel, and you are bitten by a dog. There is no need to have a booster after a dog bite (False)	44.40%	56.30%	40.70%	<0.001*
4	The bite of a healthy-looking dog/cat poses no risk of rabies (False)	72.10%	69.30%	73.00%	0.002*
5	Mean Knowledge Score (out of 12)	8.37	8.76	8.25	<0.001*

* Statistical significance.

### Factors that influenced rabies pre-exposure vaccination

Several factors including female sex, older age, longer duration of stay were found to be related with low vaccination rate. The rate of rabies vaccination also differed among travelers from different continents of origin. Travelers from North America or from Oceania had significantly lower vaccination rate when compare to travelers from Western/Central Europe while travelers from South Asia had significantly higher vaccination rate than travelers from Western/Central Europe. Details are shown in Table 3.

**Table 3:** Factor that influence rabies pre-exposure vaccination.

		Total (n)	Received at least 1 dose of vaccine		Not received vaccine		Relative Risk (95% CI)	Adjusted RR
			n	%	n	%		(95% CI)
Sex								
	Male	4771	1286	27	3485	73	1	1
	Female	2896	678	23	2218	77	0.83 (0.74–0.92)	0.77 (0.69–0.86)*
Age group								
	17–30	3529	1006	29	2523	71	1	1
	31–45	2422	618	26	1804	74	0.86 (0.76–0.97)	0.81 (0.72–0.92)*
	46–60	1307	287	22	1020	78	0.71 (0.61–0.82)	0.72 (0.61–0.84)*
	≥61	423	57	13	366	87	0.39 (0.29–0.52)	0.42 (0.31–0.56)*
Length of Stay (days)								
	0–5	2363	729	31	1634	69	1	1
	6–10	1306	292	22	1014	78	0.65 (0.55–0.75)	0.64 (0.54–0.77)*
	11–15	1163	256	22	907	78	0.63 (0.54–0.74)	0.66 (0.54–0.80)*
	16–20	678	139	21	539	79	0.58 (0.47–0.71)	0.60 (0.47–0.76)*
	>20	1917	484	25	1433	75	0.76 (0.66–0.87)	0.80 (0.66–0.95)*
Nationality								
	Western and Central European	2612	659	25	1953	75	1	1
	East Asian	2462	706	29	1756	71	1.19 (1.05–1.35)	0.98 (0.82–1.17)
	Oceania (AUS,NZ)	676	99	15	577	85	0.51 (0.40–0.64)	0.52 (0.41–0.66)*
	South Asian	543	186	34	357	66	1.54 (1.27–1.88)	1.40 (1.12–1.75)*
	North American	442	63	14	379	86	0.49 (0.37–0.65)	0.51 (0.37–0.68)*
	Middle East+Central Asian	330	93	28	237	72	1.16 (0.90–1.50)	1.08 (0.82–1.40)
	Eastern European	256	70	27	186	73	1.12 (0.83–1.48)	1.15 (0.85–1.55)
	Central and South American	180	51	28	129	72	1.17 (0.83–1.63)	1.03 (0.72–1.46)
	African	174	40	23	134	77	0.88 (0.61–1.26)	0.82 (0.55–1.20)
Reason for travel								
	Tourism	6512	1747	27	4765	73	1	1
	Business	450	80	18	370	82	0.59 (0.46–0.75)	0.57 (0.43–0.73)*
	Visiting friends and relatives	420	63	15	357	85	0.48 (0.36–0.63)	0.56 (0.42–0.74)*
	Education or research	110	28	25	82	75	0.93 (0.59–1.42)	1.07 (0.67–1.64)
	Other	158	41	26	117	74	0.96 (0.66–1.36)	0.94 (0.63–1.36)

* ***p value<0.05.***

The actual cost of rabies vaccine and its cost index, which was adjusted by the GDP per capita, differed significantly from country to country as shown in Table 4. Travelers from countries where the vaccine cost index was <20 (n = 5556) were 1.4 times more likely to receive vaccination against rabies before travel, compared to those from countries where the cost index was > = 20 (n = 2125) (27% vs. 21%, RR 1.43, 95% CI 1.27–1.61).

**Table 4:** Mean prices of cell-cultured rabies vaccine (1 dose) for pre-exposure prophylaxis in selected countries.

Country	Mean price for one intramuscular dose (USD)	Cost Index = mean price/gross domestic product per capita^1^ ×10^4^
India^2^	16	45
Sri Lanka^2^	20	40
Spain^3^	22	7
Israel	32	11
Belgium	33	9
Russia	35	18
France	38	11
Republic of Ireland	45	11
Italy	46	15
South Africa	48	46
Norway	49	9
Republic of Korea	55	19
Brazil	63	57
Japan	65	19
United Kingdom	70	19
People's Republic of China^4^	70	93
Switzerland	73	16
The Netherlands	75	18
Australia	82	21
Germany	84	22
New Zealand	101	34
Finland	110	30
Denmark	114	30
Sweden	124	32
Canada	181	47
United States of America	200	42

1 Obtained from the World Development Indicators database, World Bank, accessed 1 February 2012.

2 Prices in India and Sri-Lanka are those in private international clinics. Rabies vaccine can be obtained also from government designed anti-rabies centers in India, but almost exclusively for post-exposure prophylaxis at an average price of 8 USD per intramuscular dose. Rabies vaccine in public sector in Sri-Lanka is free and used only for post-exposure prophylaxis.

3 Price in Spain is that in private clinic. The vaccine can be obtained for free in national centers.

4 Price in the People's Republic of China is an average of prices in public and private sectors (47 USD in government designed anti- rabies centers, usually for post-exposure prophylaxis, (cost index = 62) and 93 USD in private international clinics (cost index = 123). Price in the special administrative region of Hong-Kong (77 USD, cost index = 17) was not considered is the present study.

### Risk of rabies exposure

Of 7,681 participants, sixty-six travelers (0.9%) had been bitten, while 185 travelers (2.4%) had been licked on the average stay of 23.2 days. Virtually all countries in Southeast Asia were reported as countries of exposures where travelers had been exposed to animals. The incidence of animal exposure (bitten or licked) varied from country by country ranging from 0.3% (1/325) among travelers in Malaysia to 3.6% (4/110) among travelers in Myanmar. The overall animal exposure rate in Southeast Asia was 2.8%.

Among those who were bitten, information regarding their actual practice after exposure was available in 35/66 travelers. Base on that data, 3/4 had cleaned the wound, but 2/3 did not seek medical care and did not receive post-exposure treatment. The animals most commonly encountered were dogs, followed by monkeys and cats.

Detail analysis was performed to determine risk factors that might be related to animal exposure. Age, gender, reason for travel and knowledge score had no influenced on animal exposure while the length of stay and continent of origin had some effects. Travelers from East Asia had a higher rate of exposure than Western/Central European (Adjusted RR 2.83, 95%CI 1.87–4.2). Conversely, travelers from South Asia were at lower risk (Adjusted RR 0.20, 95% CI 0.03–0.66). Apart from the nationality of travelers, the length of stay was found to be directly related with the risk of exposure. Travelers who stayed more than 20 days had a higher risk than travelers who stayed less than 5 days (5% vs 1.3%, Adjusted RR 7.78, 95%CI 4.71–13.01). Detailed of the results are show in Tables 5 and 6.

**Table 5:** Animal exposure during this trip (n = 7,681).

	no. exposed	%
Prevalence of Exposure (bitten+licked)	219	2.85
Number of travelers being bitten	66	0.86
Number of travelers being licked	185	2.41
Bitten or scratched by (n = 36)		
Dog	16	44.4
Monkey	14	38.9
Cat	3	8.3
Other	3	8.3
Among travelers who are bitten (n = 35)		
Clean the wound	26	74.3
Go to the hospital and get rabies vaccine	13	37.1
Do nothing	3	8.6

Country	Average stay (month)	Incidence of exposure*	95% CI
Thailand (n = 7681)	0.62	4.08	3.6–4.5
Cambodia (n = 633)	0.40	5.92	4.1–7.8
Vietnam (n = 520)	0.58	2.33	1.0–3.6
Lao PDR (n = 476)	0.49	4.29	2.5–6.1
Malaysia (n = 325)	0.33	0.95	0.0–2.0
Singapore (n = 276)	0.25	1.43	0.0–2.8
Indonesia (n = 183)	0.63	1.73	0.0–3.6
Myanmar (n = 110)	0.47	7.79	2.8–12.8
Philippines (n = 65)	0.54	2.85	0.0–6.9

* Incidence of exposure (bitten and licked) per 100 travelers per month of stay.

**Table 6:** Relative Risk of Animal Exposure.

	Total (n)	Exposed		Non-exposed		Relative Risk (95% CI)	Adjusted RR (95% CI)
		n	%	n	%		
Sex							
Male	4,771	132	2.8	4,639	97.2	1	1
Female	2,896	87	3.0	2,809	97.0	1.09 (0.82–1.43)	1.57 (0.78–1.39)
Age group							
17–30	3,529	118	3.3	3,411	96.7	1	1
31–45	2,422	58	2.4	2,364	97.6	0.71 (0.51–0.97)	0.84 (0.60–1.16)
46–60	1,307	32	2.4	1,275	97.6	0.73 (0.48–0.94)	0.74 (0.48–1.10)
≥61	423	11	2.6	412	97.4	0.77 (0.39–1.38)	0.61 (0.30–1.13)
Length of Stay (days)							
0–5	2,363	31	1.3	2,332	98.7	1	
6–10	1,306	24	1.8	1,282	98.2	1.41 (0.82–2.40)	2.39 (1.36–4.15)*
11–15	1,163	40	3.4	1,123	96.6	2.68 (1.67–4.33)	5.43 (3.13–9.45*
16–20	678	23	3.4	655	96.6	2.64 (1.51–4.54)	5.18 (2.76–9.60)*
>20	1,917	95	5.0	1,822	95.0	3.92 (2.64–6.00)	7.78 (4.71–13.01)*
Nationality							
Western and Central European	2,612	90	3.4	2,522	96.6	1	1
East Asian	2,462	69	2.8	2,393	97.2	0.81 (0.59–1.11)	2.83 (1.87–4.26)*
Oceania (Australian, New Zealander)	676	31	4.6	645	95.4	1.35(0.87–2.02)	1.74 (1.12–2.63)*
South Asian	543	2	0.4	541	99.6	0.10 (0.02–0.33)	0.20 (0.03–0.66)*
North American	442	13	2.9	429	97.1	0.85 (0.45–1.48)	1.05 (0.55–1.85)
Middle East+Central Asian	330	4	1.2	326	98.8	0.34 (0.10–0.83)	0.47 (0.14–1.14)
Eastern European	256	5	2.0	251	98.0	0.56 (0.20–1.25)	0.66 (0.23–1.51)
Central and South American	180	3	1.7	177	98.3	0.47 (0.12–1.28)	0.71 (0.17–1.95)
African	174	2	1.1	172	98.9	0.33 (0.05–1.04)	0.55 (0.09–1.79)
Reason for travel							
Tourism	6,512	181	2.8	6,331	97.2	1	
Business	450	13	2.9	437	97.1	1.04 (0.56–1.77)	
Visiting friends and relatives	420	15	3.6	405	96.4	1.30 (0.73–2.14)	
Education or research	110	4	3.6	106	96.4	1.32 (0.40–3.19)	
Other	158	6	3.8	152	96.2	1.38 (0.54–2.90)	
Received vaccination against rabies							
No	5,713	177	3.1	5,536	96.9	1	
Yes, only 1–2 shots	1,121	20	1.8	1,101	98.2	0.57 (0.35–0.88)	
Yes, complete 3 shots	847	22	2.6	825	97.4	0.83 (0.52–1.28)	
Knowledge score							
0–6	6,625	178	2.7	6,447	97.3	1	1
7–12	1,056	41	3.9	1,015	96.1	1.46 (1.02–2.05)	1.10 (0.75–1.58)

* *p-value<0.05.*

## Discussion

To our knowledge, this was the largest study that aimed to determine the risk of animal exposure among travelers. In our study, the risk of being bitten was 1.11 per 100 travelers per month and the risk of being licked was 3.12 per 100 travelers per month. These incidences were close to the overall estimation of risk published in one recent review. In that review, based on all available evidences [5], [10]–[13], it was estimated that 0.66% (0.02%–2.31%) of tourists will experience animal bite during one month stay [6].

It was not possible to compare our incidence rate directly with all previous studies since there were vast variations in term of the population studied, destination, definition of exposure and so on. However, several important points should be noted. Firstly, the highest incidence of animal exposure had been reported among travelers in Thailand in 1994 airport study. In that report, up to 1.3% of travelers had been bitten during an average stay of 17 days [11]. Compared to the 1994 study, our study found an approximately two-fold decrease in the risk of being bitten (1.1% per month VS 2.2% per month). The lower incidence of animal bite may result from better awareness of rabies among travelers which could by imply from the vaccination rate i.e only 1.1% of travelers in the previous study had received rabies pre-exposure prophylaxis while up to 25% of travelers in our study had received rabies vaccine before their trips.

Apart from risk of animal bite, the endemicity of rabies in the destination is also the major factor that determines the real risk of exposure to rabies virus. Fortunately, data from Thailand showed that local situation of rabies was much improved when compared to the last few decades. For example, the number of human rabies in Thailand cases had decreased from 185 cases per year in 1990 to 78 cases per year in 1994 and to less than 20 cases annually since 2001 [14]. Moreover the percentage of FAT positive animal specimens among those examined for rabies were also decline i.e. from 41% in 1990 to 28% in 2000 and to 12% in 2004 [15]. Several factors were contributed to this success such as the control of stray dogs and cats, vaccination programs in animals, mass campaigns to raise public awareness and better and more accessible post-exposure treatment [3], [14].

However it is important to note that, although the rabies situation in Thailand was much better and the risk of being bitten among travelers seemed to be lower than previous report, this risk was still high when compare to the other studies outside Southeast Asia [5], [13]. Partly, it may be due to the poor control of stray dogs and cats in many countries in Southeast Asia where more than 1 million people are estimated to be bitten annually [16]. Not only local people, but travelers in these areas are inevitably at risk also. Given that rabies is an untreatable disease once the symptoms develop, travelers in rabies endemic areas need a good basic knowledge regarding rabies risk and prevention.

Unfortunately, our study found that, travelers' attitudes and knowledge related to rabies risk were far from ideal. As seen in several previous reports [10], [17], [18], many misconceptions and misunderstandings were found among our participants, such as, up to 59% were not aware that they might get rabies after being licked by an infected animal and 50% did not know that they needed a booster vaccination once they were bitten. These misconceptions were critical and might lead to serious consequences if they actually had been exposed to the rabies virus. In our study, we also confirmed that the travelers' practice after being exposed to animal was poor i.e. one fourth of the responding travelers who were bitten had not cleaned the wound and two third of responding travelers did not go to the hospital to get a rabies vaccination. These were serious and dangerous misunderstanding. Therefore, travelers to rabies endemic areas should receive proper advice regarding rabies before their trip. Travel clinic might be a good source of information as found in several studies [10], [19], [20]. However, in our study, although travelers who had visited a travel clinic had higher mean knowledge scores than those who did not visit the clinic, some misconceptions were also found in comparable percentage between these two groups of travelers.

In this study, the length of stay in Southeast Asia was significantly related to higher rate of animal exposure. Age, gender, and travelers' knowledge, had no significant relationship to rate of animal exposure. Apart from length of stay, multivariate analysis indicated that the nationality of a traveler was related to the risk of animal exposure. Travelers from East Asia had a 2.8-fold higher risk than travelers from Western/Central Europe, while travelers from South Asia had a significantly lower risk. These differences might imply that travelers from different cultures might have different attitudes and different risk behaviors that can be related to a higher or lower risk of animal exposure. For example, travelers from South Asia where rabies was highly endemic might have higher rabies awareness than travelers from Europe, so they were less likely to risk encounter with an animal.

Through the analysis, we also found that the reason for travel was not related to the risk of animal exposure. Hence the magnitude of risk among tourists, businessmen and students in Southeast Asia could be considered the same. This finding might challenge the general belief that the activities of travelers play some role in terms of risk. Although it is logical to assume that, so far there was no available evidence to support this belief, at least in Southeast Asia. This may be in part be due to the fact that stray dogs and cats in Southeast Asia are not restricted to only certain areas, but rather can wander freely around in urban and rural areas. This might explain why, when compared to our recent study done in backpackers in Bangkok [10], the risk of being bitten in the backpacker group was even lower than that in general travelers in this study (0.69 per 100 backpackers per month VS 1.11 per 100 travelers per month). Similar findings were also reported in a study conducted in Nepal, where trekking did not increase the risk of animal exposure [5].

Although many authorities recommend pre-exposure rabies vaccination in high risk travelers [21]–[23], there was no consensus what defines “high risk”. In our study, twenty-seven percent of our participants received rabies vaccine before their trips. Several factors including male sex, younger age, travel for tourism and, surprisingly, a shorter length of stay were found to be correlated to higher vaccination rates. We also found that travelers from countries with a cost index <20 were more likely to receive the vaccine. As in many studies, this was confirmed that cost of the vaccine was an important factor that travelers consider before receiving the pre-exposure vaccines [10], [24], [25].

Our study had some limitations. Although we surveyed more than 7,000 departing travelers from Suvarnabhumi International Airport, which is the main airport hub in Southeast Asia, data from a single airport is not ideal for representing the whole of Southeast Asia. Our data should strongly represent travelers in Thailand and its neighboring countries such as Lao PDR, Cambodia and Vietnam, because most of them use Suvarnabhumi International Airport as a travel hub. But our data may underrepresent people who travel mainly in Indonesia, Singapore and the Philippines, since they may use other airports. Ideally, a multi-airport study could provide more comprehensive data.

Second, the language barrier may have led to selection bias in our study. In this study, apart from English, we translated our questionnaire to 3 different languages i.e. Chinese, Japanese, and Korean. However, the questionnaire were not translated into Arabic, Hindi, Spanish, or any African languages. So those travelers from the Middle East, India, Africa and Latin America, who did not understand English, had to be excluded from the study. It is possible that travelers from those areas who understood English and those who did not may have different risk characteristics.

Third, children, who represent a recognized at-risk population for animal bites and rabies, [1], [2] were not included in our survey, which may have biased the results.

We could conclude that travelers in Southeast Asia, regardless of their reasons for travel, had a significant risk of being bitten or licked by animals while traveling. A longer duration of stay was associated with a higher risk. However, it must be pointed out that 53.8% of travelers with exposure to potential rabies infected animals were actually exposed while traveling for less than 3 weeks. Many were inadequately informed and lacked a basic knowledge of this life-threatening risk. Rabies prevention advice should be included in every pre-travel visit.
